# Influence of PEGDA Molecular Weight and Concentration on the In Vitro Release of the Model Protein BSA–FITC from Photo Crosslinked Systems

**DOI:** 10.3390/pharmaceutics15041039

**Published:** 2023-03-23

**Authors:** Natalia Rekowska, Katharina Wulf, Daniela Koper, Volkmar Senz, Hermann Seitz, Niels Grabow, Michael Teske

**Affiliations:** 1 Institute for Biomedical Engineering, University Medical Center Rostock, Friedrich-Barnewitz-Straße 4, 18119 Rostock, Germany; 2 Chair of Piston Machines and Internal Combustion Engines, University of Rostock, Albert-Einstein-Straße 2, 18059 Rostock, Germany; 3 Institute for Implant Technology and Biomaterials E.V., Friedrich-Barnewitz-Straße 4, 18119 Rostock, Germany; 4 Microfluidics, Faculty of Mechanical Engineering and Marine Technology, University of Rostock, Justus-von-Liebig-Weg 6, 18059 Rostock, Germany; 5 Department LL&M, Interdisciplinary Faculty, University of Rostock, Albert-Einstein-Str. 25, 18059 Rostock, Germany

**Keywords:** drug delivery systems, 3D printing, photopolymerization, PEGDA, BSA–FITC, drug release

## Abstract

Novel 3D printing techniques enable the development of medical devices with drug delivery systems that are tailored to the patient in terms of scaffold shape and the desired pharmaceutically active substance release. Gentle curing methods such as photopolymerization are also relevant for the incorporation of potent and sensitive drugs including proteins. However, retaining the pharmaceutical functions of proteins remains challenging due to the possible crosslinking between the functional groups of proteins, and the used photopolymers such as acrylates. In this work, the in vitro release of the model protein drug, albumin–fluorescein isothiocyanate conjugate (BSA–FITC) from differently composed, photopolymerized poly(ethylene) glycol diacrylate (PEGDA), an often employed, nontoxic, easily curable resin, was investigated. Different PEGDA concentrations in water (20, 30, and 40 wt %) and their different molecular masses (4000, 10,000, and 20,000 g/mol) were used to prepare a protein carrier with photopolymerization and molding. The viscosity measurements of photomonomer solutions revealed exponentially increasing values with increasing PEGDA concentration and molecular mass. Polymerized samples showed increasing medium uptake with an increasing molecular mass and decreasing uptake with increasing PEGDA content. Therefore, the modification of the inner network resulted in the most swollen samples (20 wt %) also releasing the highest amount of incorporated BSA–FITC for all PEGDA molecular masses.

## 1. Introduction

Drug delivery systems (DDS) function as medical products that introduce a pharmaceutically active agent systemically or locally to the body in a highly controlled manner. In comparison with traditional enteral or parenteral routes of administration, they ensure the effectiveness and safety of the treatment with minor side effects. Furthermore, the application of DDS offers various possibilities regarding individual patient-tailored pharmaceutical therapies that are optimal for various persons and medical purposes. Although many approved DDSs have been successfully implemented as medications, recent advances in this research field reveal the great potential of this approach in the pharmaceutical sciences [1,2,3,4].

Among different techniques, 3D printing technologies enable the production of digitally designed, personalized, and complex DDS scaffolds that can also be precisely crafted as on-demand products [5,6,7]. In the future, these techniques should facilitate THE fabrication of numerous constructs with variable doses and release profiles specified for a particular person. They should also enable production of multidrug DDSs, which are difficult to achieve in traditional dosage formulations. Examples of such 3D-printed, multisubstance preparations can be found in the literature [8,9]. These flexible techniques are also studied as a tool for the preparation of medications for unique groups such as children [10] and patients requiring local treatment such as cancer therapy to avoid broad systemic side effects [11].

A common and promising 3D printing technique is stereolithography, employing photopolymerization in the manufacturing process, and it is versatile, cost-effective, and rapid [12,13,14,15]. In this method, drug carriers are hydrogels that are prepared via the solidification of photopolymers via free-radical-initiated chain polymerization reaction. Briefly, in the presence of a light source and a photoinitiator (PI), free radicals are formed. These excited molecules react with the acrylate or vinyl groups of the photopolymers, resulting in covalent crosslinking between polymer chains [16,17].

Stereolithography is a tool that allows for both the creation of personalized medication and the effective administration of very attractive and potent, but also challenging therapeutic agents such as proteins. Proteins are susceptible to protease degradation and other inactivating factors under physiological conditions. This is why alternatives to traditional routes of administration, enhancing their bioavailability, such as drug delivery systems are intensively investigated [18,19,20]. Stereolithography is also a gentle and accurate curing technique for DDSs releasing thermolabile substances such as proteins and should be thoroughly investigated in this context [21]. Understanding the factors influencing the photopolymerization process and the characteristics of a polymer material is crucial for the design of novel, patient-tailored, highly controllable DDS devices [19]. Most of the studies employing stereolithography as a DDS preparation method focused on the incorporation of small, synthetic molecules without photo-cross-linkable groups such as ibuprofen, paracetamol, aspirin, ketoprofen, caffeine, or prednisolone [22,23,24].

In this study, we investigate the well-established photopolymerizable poly(ethylene) glycol diacrylate (PEGDA), differing in molecular mass (4000, 10,000, and 20,000 g/mol), as a carrier for model protein drug albumin–fluorescein isothiocyanate conjugate (BSA–FITC). BSA–FITC was used as the model drug because it has a similar molecular mass and structure to those of bone morphogenetic protein (BMP-2), and exhibits similar binding affinity to collagen type as that of some other growth factors [25,26]. PEGDA, as a biocompatible and hydrophilic compound, is often studied for different biomedical applications such as 3D printing techniques [25,26,27,28]. Moreover, examples of PEGDA scaffolds as DDS incorporating peptide active agents were reported [29,30]. Loading a protein through matrix swelling is a gentle process that does not affect the protein structure. Preparing protein-releasing DDSs with methods employing photopolymerization remains challenging due to the possible crosslinking between the reactive groups of the polymer, such as acrylate and vinyl groups, and proteins, such as amino and sulfhydryl groups [31,32]. Previous studies showed that the modulation of the inner structure of the PEGDA network via the combination of different monomer masses fundamentally impacts the biophysical properties of the scaffold and could crucially influence water diffusion rates [27,33]. This indicates that altering the molecular mass and polymer concentration in the matrix determines the mechanical hydrogel properties such as softness and protein drug release via diffusion through the scaffolds [34]. Therefore, here, we investigate the in vitro BSA–FITC release to present the drug release profiles of differently composed matrices. BSA–FITC was chosen as a model drug due to the high sensitivity of fluorescent quantification [35,36]. The intramolecular quenching effect that alters the fluorescent emission and is characteristic for fluorescently labeled ligands can be overcome with a simple proteolytic procedure [37]. We also analyzed other important hydrogel characteristics such as the swelling ratio and thermal behavior of the prepared DDS. Additionally, the rheological behavior of the unpolymerized photopolymers was examined. Such considerations are essential to recognize the potential of the generated biomaterial for particular medical applications, and are necessary to predict their compatibility with 3D printing devices.

## 2. Materials and Methods

### 2.1. Materials

Poly(ethyleneglycol) diacrylate M_w_ = 4000 g/mol (PEGDA_4000_), poly(ethyleneglycol) diacrylate M_w_ = 10,000 g/mol (PEGDA_10000_), poly(ethyleneglycol) diacrylate M_w_ = 20,000 g/mol (PEGDA_20000_), pronase from *Streptomyces griseus*, albumin–fluorescein isothiocyanate conjugate (BSA–FITC), photoinitiator (PI) lithium phenyl-2,4,6-trimethylbenzoylphosphinate (LAP), and buffer components N-[tris(hydroxymethyl)methyl]-2-aminoethansulfonsäure (TES), NaCl, KCl, and CaCl2 were all purchased from Merck KGaA (Darmstadt, Germany).

### 2.2. TES Buffer Preparation

The TES buffer was prepared by dissolving 30 mM TES, 140 mM NaCl, 4 mM KCl, and 10 mM CaCl_2_ in purified water, and adjusting the pH to 7.5.

### 2.3. PEGDA Polymerization

Samples were prepared from 20, 30, or 40 wt % of PEGDA_4000_, PEGDA_10000,_ or PEGDA_20000_ in water/methanol (1:2) (*w/w*) solutions. Each sample contained 0.5% (*w/w*) LAP as PI and 0.075% (*w/w*) of BSA–FITC as the model drug (both referring to PEGDA amount).

Samples were prepared in a silicone holder that we produced to form cylinder samples (Ø = 6 mm, h = 1 mm). The solutions were carefully pipetted into the wells of the silicone holder on the laboratory scales (KERN 770, Frommern, Germany) to ensure the same polymer amount in each sample. The samples were polymerized in the UV chamber (CL-1000L, UVP, Upland, CA, USA) at λ = 365 nm for 10 min, and dried for 24 h in the vacuum chamber.

### 2.4. Morphology Analysis

Scanning electron microscopy (SEM) images were obtained with the use of Quanta FEG 250 (FEI GmbH, Dreieich, Germany) under 50 Pa and 3 kV. A secondary Everhart–Thornley electron detector (ETD) was used. Image magnification ranged from 50× to 1000×.

### 2.5. Differential Scanning Calorimetry

Thermal analysis was carried out with a DSC1 (Mettler Toledo GmbH, Greifensee, Switzerland) under a nitrogen purge. High-purity indium and zinc were used for temperature calibration, and an indium standard was used to calibrate the fusion heat (Δ*H*). The weights of the samples ranged from 10 to 20 mg. We used the −50 → 200 → −50 → 250 °C temperature profile for the measurements with a heating rate of q = 10 K/min (n = 3). The data were analyzed with respect to melting temperature (*T_m_*). Due to the focus on the drug release properties of the generated systems, we used the first heating cycle for analysis.

### 2.6. Swelling Behavior Evaluation

The swelling behavior of the hydrogels was studied in the TES buffer at 37 °C. Disk samples (Ø = 6 mm) were weighed before and after 24 h of swelling. The swelling behavior was tested separately 3 times for each hydrogel (n = 3).

### 2.7. Rheological Measurements

The viscosity of 20, 30, and 40 wt % of PEGDA_4000_, PEGDA_10000_ and PEGDA_20000_ dissolved in water/methanol (1:2) without the addition of the model drug and PI was characterized using rotary rheometer Haake Rheostress 1 (Thermo Scientific, Karlsruhe, Germany) and a 1° cone with plate geometry. For the applied shear rates, a gradient from 100 to 300 s^−1^ shear stress of each PEGDA solution was measured at 15 °C (n = 3), and the viscosity values were calculated from the Newtonian conditional equation by dividing shear stress τ by the corresponding shear rate γ.

### 2.8. In Vitro Release of BSA–FITC

The in vitro drug release of BSA–FITC was separately conducted for each sample (Ø = 6 mm, h = 1 mm) in 1 mL TES at 37 °C and shaking with 100 rpm in the dark. The release was performed for over 1032 h (43 days) with complete medium exchange at particular time points. In order to avoid the quenching effect of FITC, the release was followed by the digestive procedure with pronase described by Breen et al. [37]. Briefly, each medium sample was treated with pronase from Streptomyces griseus (100 µg per sample) and incubated in the dark for 72 h before fluorescent determination at 37 °C. The residual release of BSA–FITC was determined by solving the sample in formic acid, followed by freeze-drying to remove the acid and the uptake of the residues in TES and the described digestion by pronase procedure. Standard calibration solutions of BSA–FITC were prepared in TES.

The fluorescent BSA–FITC determination was performed in black 96-well plates (Greiner Bio-One 655086, Frickenhausen, Germany) with Fluostar Optima (BMG LABTECH, Ortenberg, Germany), with excitation at 485 nm and emission at 520 nm. Mean values (MVs) and standard deviations (SDs) were calculated from n = 5 samples.

### 2.9. Statistical Analysis

Statistical differences were determined with one-way analysis of variance (ANOVA) that was followed by multiple-comparison procedures (Holm Sidak method) provided by SigmaPlot (Systat Software Inc., San Jose, CA, USA). *p* values < 0.05 indicated significant differences.

## 3. Results

### 3.1. Rheological Behavior

**Figure 1:**
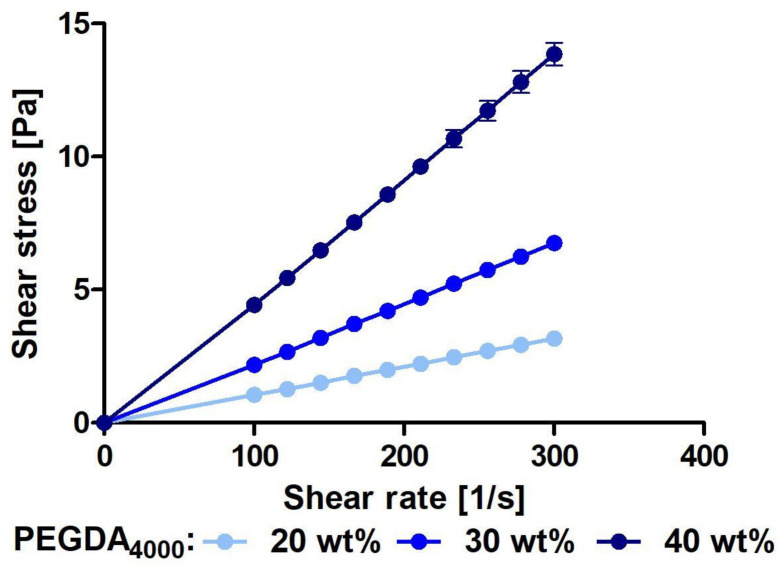
Exemplary shear stress/shear rate representation of PEGDA_4000_ solutions in H_2_O/MeOH (1:2) with different concentrations of the polymer at 15 °C (n = 3).

The rheological behavior of unpolymerized pure PEGDA water/methanol (1:2) solutions was measured. The relationship between the shear stress and the shear rate is illustrated in Figure 1. A linear increase in shear stress with increasing shear rate was observed for all of polymer concentrations, meaning that all materials exhibited Newtonian behavior. This behavior was also observed for PEGDA_10000_ and PEGDA_20000_ (Supplementary Figures S1 and S2).

**Figure 2:**
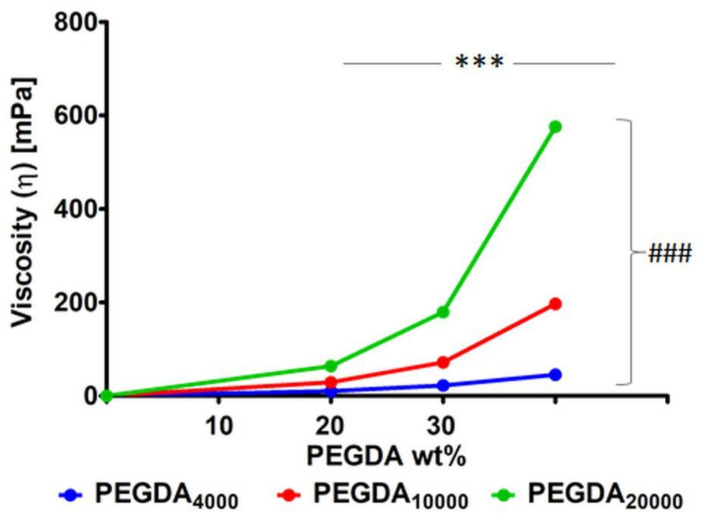
Average viscosity (mPa·s) for unpolymerized PEGDA solutions with different concentrations of the polymer in H_2_O/MeOH (1:2) at 15 °C (n = 3). There were significant differences for all of the samples, marked with *** for differences between different wt % of the same polymer, and with ### for differences between the same wt % of the same polymer (*p* < 0.001).

The average dynamic viscosity for each PEGDA solution was calculated and is presented in Figure 2. The viscosity of the samples increased with the increasing concentration of the polymer in the sample. This trend is remarkable, especially in the case of PEGDA_20000_, where viscosity increased from 64 mPa·s for the 20 wt % solution to 576 mPa·s for the 40 wt % solution, which corresponds a 900% increase in viscosity. In the case of PEGDA_10000,_ it was a 679% increase, and a 409% increase for PEGDA_4000_. Significantly increased values of the viscosity were also observed for samples with a higher molecular mass. Here, the highest discrepancies were observed for the 40 wt % samples: 64 s, 179, and 575 mPa·s for PEGDA_4000_, PEGDA_10000_ and PEGDA_20000_, respectively.

### 3.2. Surface Morphology

**Figure 3:**
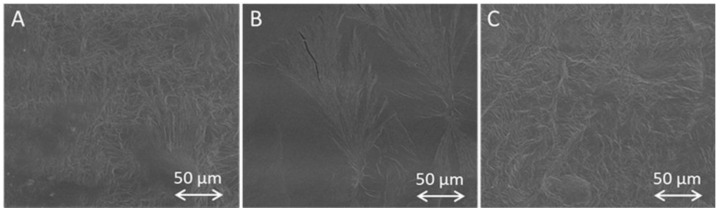
Comparison of representative SEM images for PEGDA samples (20 wt %) with different molecular masses of (**A**) 4000, (**B**) 10,000, or (**C**) 20,000 g/mol containing BSA–FITC.

The surface morphology of all investigated samples (PEGDAs 4000, 10,000, and 20,000 g/mol) was characterized with SEM. The PEGDA sample surfaces showed no visible changes by changing the molecular mass of the polymer (Figure 3). Increasing the polymer concentration in the sample (20, 30, and 40 wt %) also did not introduce any changes in the surface morphology, with the only exception of the PEGDA_4000_ 40 wt % samples, of which the surface was more structured (Figure S3). Moreover, the PEGDA surface morphology was unaffected by the incorporation of BSA–FITC as the model drug (Figure S4).

### 3.3. Thermal Properties

**Figure 4:**
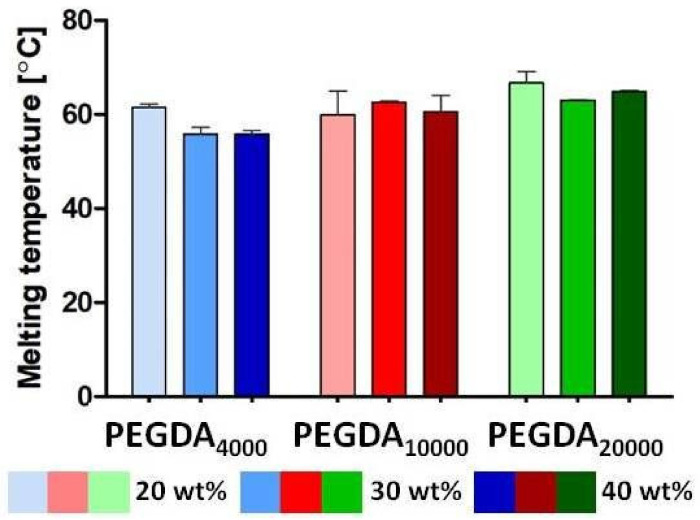
Mean melting temperature (°C) ± SD for PEGDA_4000_, PEGDA_10000,_ and PEGDA_20000_ samples containing 20, 30, or 40 wt % of the polymer and loaded with 0.075 wt % BSA–FITC (referring to PEGDA amount, n = 3).

Figure 4 shows the melting temperature *Tm* of the PEGDA hydrogels with various molecular masses in different concentrations. The tested samples showed no trends in *Tm* with increasing polymer concentration. In contrast, increasing the molecular weight of the monomers increased *Tm* for the same polymer concentrations. However, these differences were not significant, and only a tendency was detected. Thermal behavior was unaffected by the addition of model drug BSA–FITC (Figure S5).

### 3.4. Swelling Behavior

**Figure 5:**
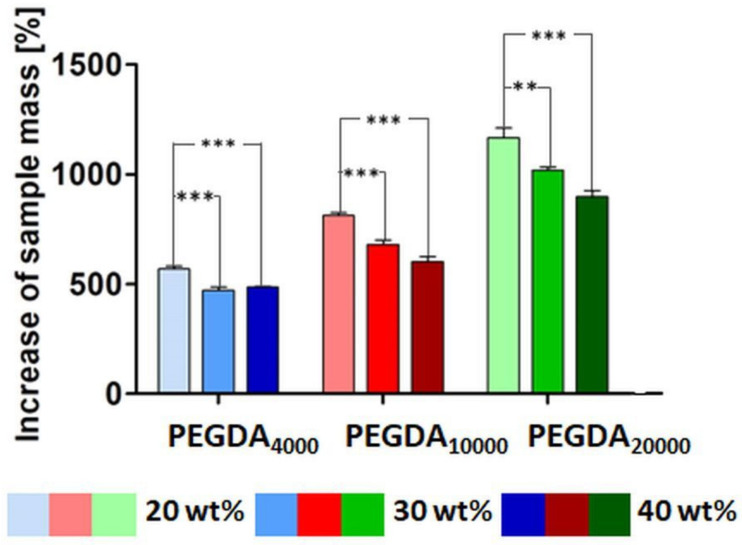
Increase in sample weight (samples with BSA–FITC) in percentages after 24 h swelling in a TES buffer at 37 °C (n = 3). Significant decreases in weight growth between the 20 wt % samples and higher concentrations are marked with ** for *p* < 0.01 and *** for *p* < 0.001. Significant differences in weight between the same concentration of different molecular masses of PEGDA occurred for all of the samples (*p* < 0.001) and are not marked on the graph.

The swelling behavior of PEGDA hydrogels with different molecular masses and their concentration are shown in Figure 5. The increase in the molecular mass of PEGDA resulted in a significantly increased amount of the absorbed medium (*p* < 0.001). This trend was especially distinct in the 20 wt % samples. After 24 h of swelling in the TES buffer, the mass of the samples containing 20 wt % of PEGDA_4000_ increased by about 6 times; in the case of PEGDA_10000,_ it was over 8 times, and for PEGDA_20000_, it was about 12 times. A similar tendency with a slightly lower absorbed medium amount, but with significant differences, was observed for the samples containing 30 and 40 wt % of the polymer. Here, only the 30 wt % PEGDA_4000_ samples exhibited a discrepancy and took up less of the medium than the PEGDA_4000_ 40 wt % samples did; this difference was not significant.

**Figure 6:**
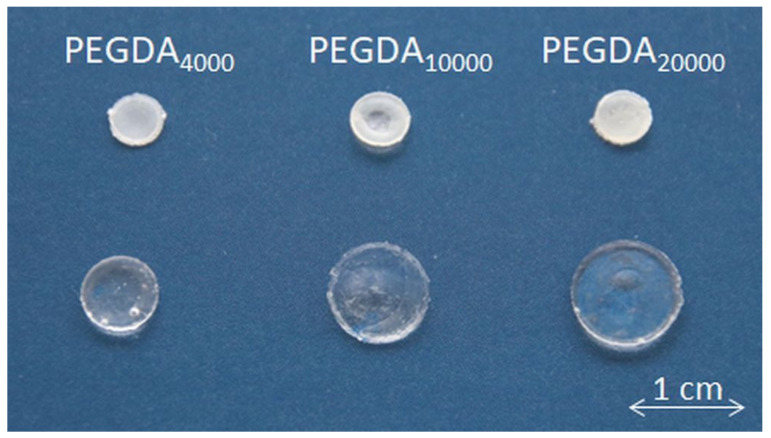
Macrophoto of exemplary samples PEGDA_4000_, PEGDA_10000,_ and PEGDA_20000_ with 40 wt % polymer content before (**top**) and after (**bottom**) 24 h of swelling in a TES buffer at 37 °C.

Exemplary samples containing 40 wt % of PEGDA before and after the test are illustrated in Figure 6. The depicted results refer to samples containing BSA–FITC as the model drug. Results for the pure PEGDA samples were similar and can be found in Supplementary Figure S6.

### 3.5. In Vitro Drug Release

**Figure 7:**
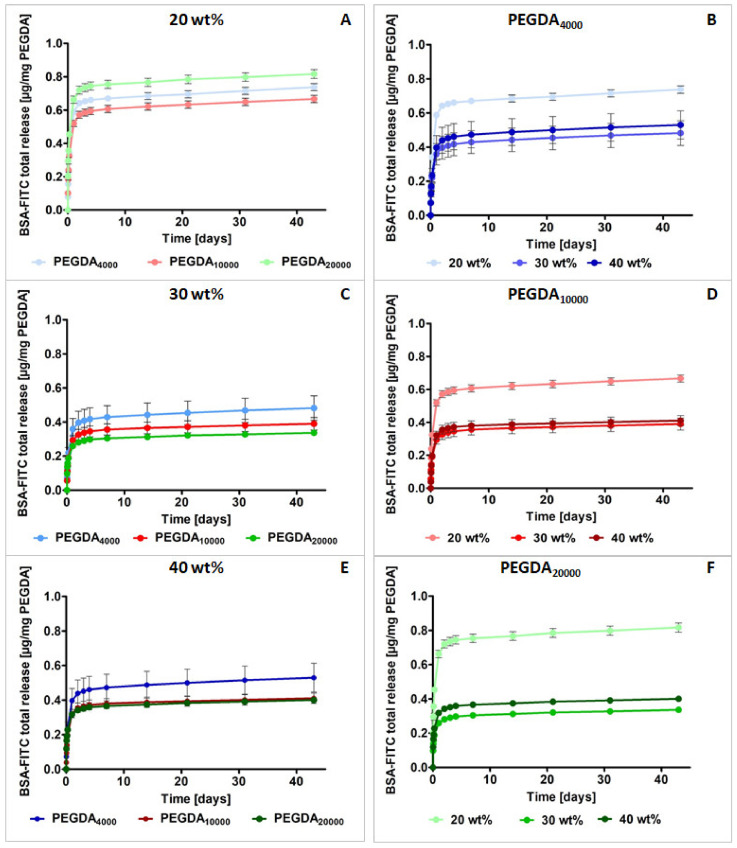
Total quantitative release of BSA–FITC within 43 days normalized to 1 mg PEGDA in mean ± SD. (**left**) Different PEGDA concentrations: (**A**) 20 wt %; (**C**) 30 wt %; (**E**) 40 wt %. (**right**) PEGDAs with different molecular mass: (**B**) PEGDA 4000 g/mol; (**D**) PEGDA 10,000 g/mol; (**F**) PEGDA 20,000 g/mol. Each sample contained 0.075 wt % of BSA–FITC (referring to PEGDA amount). Release occurred in 1 mL of a TES buffer at 37 °C by shaking at 100 rpm in the dark (n = 5).

The release of model drug BSA–FITC from the PEGDA samples is shown in Figure 7. In order to compare the release curves regarding PEGDAs with a different molecular mass (Figure 7A,C,E) and polymer weight (Figure 7B,D,F), polymer and BSA–FITC concentrations were kept constant for all samples. The total release of BSA–FITC was normalized to 1 mg of PEGDA in the samples to compensate for the differences in sample weight. PEGDA_4000_ samples released the most BSA–FITC within 43 days of the experiment: on average, 0.58 µg BSA–FITC/mg PEGDA_4000_ (C and E). In the case of PEGDA_10000_, it was 0.48 µg/mg PEGDA, and for PEGDA_20000_, it was 0.53 µg/mg PEGDA. Only samples containing 20 wt % of the PEGDA_20000_ polymer demonstrated a higher release than that of the other specimens with 20 wt %.

For all analyzed molecular masses, 20 wt % of PEGDA samples had the highest BSA–FITC release in comparison with that of other concentrations. Differences between the 30 and 40 wt % samples for all of PEGDAs were rather negligible, ranging between 0.02 and 0.06 µg of the released BSA–FITC/mg PEGDA. All BSA–FITC release profiles were comparable in shape, with an initial burst release within the first 2 days.

## 4. Discussion

Our investigation, characterizing PEGDA as a potential material for 3D photochemical DDS applications, was designed to keep the wt % of all of the components (polymer and BSA–FITC) in the samples equal for all compared specimens in order to analyze the influence of the polymer concentration (20, 30 and 40 wt %) and molecular mass of the used PEGDA (4000, 10,000, and 20,000 g/mol).

**Figure 8:**
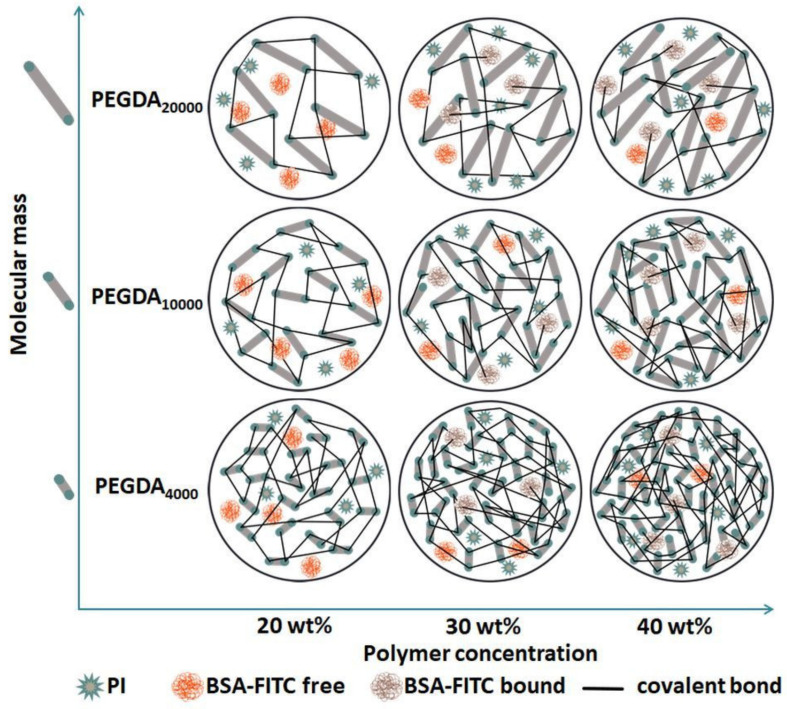
Schematic illustration of hydrogel network formed with the photopolymerization of differently concentrated PEGDA_4000_, PEGDA_10000_, and PEGDA_20000_ solutions with PI (stars) and BSA–FITC (balls). PEGDA is represented as sticks connected into longer chains with covalent bonds.

The possible reasons and explanations for the in vitro BSA–FITC release presented here are summarized in Figure 8, which shows the hydrogel network formed with the photopolymerization of differently concentrated PEGDA_4000_, PEGDA_10000,_ and PEGDA_20000_ solutions, and the possible resulting differences.

Studies on the mechanics of the analyzed systems were published [38]. In summary, the samples’ elongation capacity (range: ~8 to ~958%) clearly increased with increasing molecular mass, whereas increasing the PEGDA concentration resulted in significantly higher tensile strength (range: ~0.2 to ~13 MPa). The mechanical properties could lead to applications as a drug delivery system for soft tissue with low mechanical stress, tissue engineering, or as coatings due to their swelling behavior, discussed later.

In addition to focusing on the release of model protein BSA–FITC, our earlier studies also considered the biocompatibility of similar PEGDA material systems [28]. As a result, thorough rinsing to remove water-soluble toxic photoinitiators or low-molecular-weight residues is mandatory. The swelling of PEGDA facilitates rinsing with aqueous solutions. The loss of covalently bound active agent BSA–FITC during rinsing is unlikely, but possible changes in mechanics must be taken into account.

### 4.1. Swelling Behavior

Many factors, including different physical and chemical forces, influence the water uptake and swelling behavior of hydrogels [28]. For instance, entanglements, the presence of crystallites, and crosslinks significantly hinder water absorption [39]. Samples prepared from PEGDA with a higher molecular mass absorbed more of the medium (4000 < 10,000 < 20,000). Similar observations were reported before [40,41]. Most likely, the increased molecular mass clearly decreased the number of free acrylate groups, which are able to form covalent crosslinks (Figure S7). This resulted in a lower crosslinking degree (Figure 8) and higher elastic response of the PEGDA chains, and a higher amount of water that could be absorbed [41,42]. The molecular weight, length, and mobility of the monomer chains is an important factor affecting photopolymerization and thereby the resulting crosslinking degree [43]. The longer the chains are, the more restricted their mobility is. Mobility decreases even more during the photopolymerization process and hinders the chains’ migration towards the radical groups [44]. This is another factor that can lead to fewer covalent crosslinks and greater mesh size, which increases the uptake of water [42]. A significantly decreased amount of the absorbed medium in samples containing more polymers can be explained by the fact that the higher density of the polymer chains in the matrix reduced the diameter of the pores between them (smaller mesh, schematically shown in Figure 8), which decreases the water uptake in hydrogels [45]. The exceptions were the PEGDA_4000_ 30 and 40 wt % samples, where no difference was observed. We assumed that, although there were more available acrylate groups (Figure S7) in the PEGDA_4000_ 40 wt % solution, the fast process of photopolymerization led to a rapid decrease in PEGDA chain mobility and the termination of the reaction [44,45].

### 4.2. Viscosity of PEGDA Solutions

The viscosity characterization of biomaterials employed in stereolithography is largely studied as one of the crucial factors in choosing the resin for a 3D printing process [46]. The high viscosity of the material negatively affects the polymerization and conversion rates of reactive C=C double bonds due to the decreased mobility of the monomer molecules in the reacting solution [47,48]. In addition, processability in various lithographic 3D printing processes significantly depends on the viscosity of the resins. In general, low viscosity is advantageous in laser-based stereolithography and digital light processing (DLP). In laser-based stereolithography, high viscosities can lead to problems during recoating, as the generation of thin resin layers becomes increasingly difficult due to poor flowability [49]. A limit of 3 Pa·s viscosity was reported in the literature in the context of ceramic slurries [50]. In a typical DLP process, the build surface is illuminated from below via a glass window, eliminating the recoating step. The increase in viscosity alters the flow dynamics of the resin, and affects the wetting mechanism of the build window, increasing the mechanical force required to lift the build platform [51]. In the DLP process, a viscosity limit of 3 Pa·s was mentioned in the literature in connection with ceramic-loaded slurries [52]. In the literature, fixed viscosity limits are rarely mentioned, since the limit value, especially for particle-laden slurries, must be determined individually for each material, and depends on the specific stereolithography or DLP equipment and the tolerable loss of component quality caused by increased viscosity.

Among the investigated materials in this study, the polymer with the highest molecular mass, PEGDA_20000_, showed the highest viscosity of all used concentrations (Figure 2). These observations are consistent with the existing literature reporting that higher molecular mass results in impaired chain mobility. This, in turn, increases flow resistance. The relatively small differences between the 20 wt % solutions drastically grew with the increasing concentration of the polymer in the solution. This means that, for PEGDA, the viscosity increased exponentially with increasing molecular mass. This also indicates that PEGDA’s molecular mass and concentration both increased the viscosity of the solution, the former by decreasing chain mobility through the higher chain length, and the latter because of the increasing number of particles in the solution. However, since the viscosity values of all investigated PEGDA compositions were far from the limit of 3 Pa·s, it could be assumed that the material could be processed on many common laser-based stereolithography and DLP systems [50,52]. Should there still be problems with 3D printing due to high viscosity, resins could be processed at elevated temperatures. Alternatively, infrared (IR) lamps can be used as a heat source [53].

### 4.3. Surface Morphology

Analysis of the sample surface did not reveal any clear variations between the drug-loaded and pure PEGDA samples. All of the investigated specimens had a slightly structured and nonuniform appearance. Therefore, the addition of low concentrations of model protein BSA–FITC did not strongly impact the morphology during photopolymerization.

### 4.4. Thermal Properties

Drug incorporation did not have a significant influence on the *Tm* of the investigated polymerized samples. The increase in the PEGDA’s molecular mass slightly increased *Tm*. The phenomenon of an increased *Tm* via increasing the molecular mass of the used monomers was described in the literature [54,55]. This is explained by the fact that an increase in chain length results in fewer free acrylate end groups; therefore, the mobility of the end (acrylate) groups is also limited, which increases *Tm* values [56,57]. Therefore, the high differences in the molecular weight of the monomers had negligible influence on the Tm of the generated networks.

### 4.5. In Vitro Drug Release

All of the samples showed similar curves, with an initial burst release within the first 2 days followed by a slower release of the last 10–15% of BSA–FITC within the next 40 days (Figure 7).

The highest absolute release of BSA–FITC could be observed for all 20 wt % samples. In these samples, as described in Section 4.1, the highest uptake of the medium was also observed. These observations are consistent with the existing literature where the material degree of the swelling was reported as one of the factors significantly influencing the in vitro drug release, and higher water absorption was related to higher drug release [58]. Consequently, water diffusion and the elution of the model drug were facilitated.

However, next to water diffusion, the formation of covalent bounds between BSA–FITC and PEGDA during the photopolymerization process may be an important aspect in the release of proteins from polymer matrices. PEGDA particles, instead of reacting only with each other, also covalently bound the incorporated BSA–FITC under formation of hydrolysable ester bonds with functional groups such as –NH2 or –SH [59]. A lower PEGDA concentration most probably results in less crosslinked BSA–FITC and more unbound BSA–FITC. Thus, from the samples containing higher PEGDA, substantially lower amounts of unbound BSA–FITC per 1 mg PEGDA could be released (Figure 7). This explanation was confirmed via the released BSA–FITC amounts from all 30 and 40 wt % PEGDAs, which were lower than those for the 20 wt % PEGDA. The reduction in released BSA–FITC amounts between the 20 and 30 or 40 wt % samples of PEGDA increased with increasing molecular mass. Compared to the 20 wt % PEGDA, 30 wt % PEGDA_4000_ released 35% less BSA–FITC; for PEGDA_10000_, it was 42% less, and for PEGDA_20000_, it was 61 wt % less (Figure S8). Furthermore, this effect could possibly be related to the higher viscosity of the 30 and 40 wt % PEGDA solutions, which resulted in the reduced movability of PEGDA monomer chains and the hindered crosslinking.

Surprisingly, the released amount of BSA–FITC from all 30 and 40 wt % PEGDAs was relatively similar. Thus, for 30 and 40 wt % PEGDAs, no significant reduction in the released BSA–FITC amounts was detected for the different molecular masses and consequently the different viscosities (Figure 7). This happened even though the higher concentration of the polymer was equivalent with an increased number of reactive acrylate groups (Supplementary Figure S7). In the case of concentrations up from around 30 wt % of PEGDA, the amount of the bound or trapped BSA–FITC did not clearly change, even though the differences in viscosity drastically increased for the 30 and 40 wt % samples, so viscosity up from 30 wt % probably did not influence the formation of crosslinks during the curing any more (Figure S9). In addition to the influence of crosslinking on the release and stability of BSA–FITC, UV light irradiation must also be taken into account. We assumed that its influence was small, since the sample polymerized quickly and a UV light-absorbing polymer was thereby formed at the surface. The choice of PI and light source also needs to be further optimized and tested for applications in stereolithography.

Apart from crosslinking, the release behavior could be affected by generating copolymers or blend compositions (not used in this work), or the degradation rate of the matrix [60]. Nevertheless, PEGDA is a slow degrading polymer; thus, the influence of degradation on drug release is probably negligible [61].

## 5. Conclusions

There has been much attention focused on achieving the sustained release of a drug, and thereby a better and more controlled therapeutic effect [62]. Photoresins such as PEGDA could be taken into consideration as protein carriers for the development of DDS resins for novel 3D printing techniques. Knowledge about its properties, such as viscosity and the resulting crosslinking structure, is crucial in choosing the best printable composition of the resins to possibly crosslink and release the drug as desired.

In this work, the influence of the PEGDA composition on the release of model protein drug BSA–FITC was studied. This systematic study revealed that PEGDA concentration and molecular mass have an unambiguous influence on its viscosity. These affect the mechanism of photopolymerization and the formation of covalent bonds between reacting photomonomers and protein drugs, and the swelling behavior of the resulting 3D print. The analysis of the release outcomes showed that the factors influencing drug release are complex, and at the higher concentrations of PEGDA, no simple correlations among viscosity, water uptake, and the released protein could be found, which leaves room for interpretation and needs further study. However, between 20 and 30 wt %, factors such as swelling behavior and viscosity had the highest impact on the crosslinking between the different components in the system and on the in vitro BSA–FITC release. Within this range, drug release may be highly tunable, which indicates that accurate investigations such as ours are challenging, but also essential and required in understanding designing drug depots in 3D printing such as stereolithography. Further investigations of crosslinking and the adjustment of resin properties for inkjet use and exposure parameters are necessary.

## Data Availability

Not applicable.

## Supplementary Materials

The following supporting information can be downloaded at: https://www.mdpi.com/article/10.3390/pharmaceutics15041039/s1. Figure S1: exemplary shear stress/shear rate representation of PEGDA_10000_ solutions in H_2_O/MeOH (1:2) with different concentrations of the polymer at 15 °C (n = 3); Figure S2: exemplary shear stress/shear rate representation of PEGDA_20000_ solutions in H2O/MeOH (1:2) with different concentrations of the polymer at 15 °C (n = 3); Figure S3: comparison of SEM images of the surface of PEGDA samples with different molecular masses (4000 g/mol, 10,000 g/mol and 20,000 g/mol) and their concentrations with BSA–FITC; Figure S4: comparison of SEM images of the surface of PEGDA samples with different molecular masses (4000 g/mol, 10,000 g/mol and 20,000 g/mol) and their concentrations without BSA–FITC; Figure S5: mean melting temperature (°C) with SD for PEGDA_4000_, PEGDA_10000_, and PEGDA_20000_ samples containing 20, 30, or 40 wt % of the polymer without BSA–FITC (n = 3); Figure S6: increase in sample (Ø = 6 mm, thickness = 1 mm) weight (pure PEGDA without BSA–FITC) in percentages with SD after 24 h swelling in a TES buffer at 37 °C (n = 3); Figure S7: theoretical number of free acrylate groups in 1 mL of unpolymerized of different PEGDA solutions; Figure S8: differences in percentage between the 20–30 and 20–40 wt % PEGDA samples in the released amount of BSA–FITC; Figure S9: relationship between total BSA–FITC release and PEGDA viscosity (4000, 10,000, and 20,000 g/mol) solutions (20, 30, and 40 wt %).
