# Supplementary material for: Influence of PEGDA Molecular Weight and Concentration on the In Vitro Release of the Model Protein BSA–FITC from Photo Crosslinked Systems
Source: Pharmaceutics. 2023 Mar 23;15(4):1039. doi: 10.3390/pharmaceutics15041039 (PMC10145661; doi:10.3390/pharmaceutics15041039)
Supplement: Supplementary file 1 [file pharmaceutics-15-01039-s001.zip › pharmaceutics-2258268-supplementary.pdf]

Supplementary Material

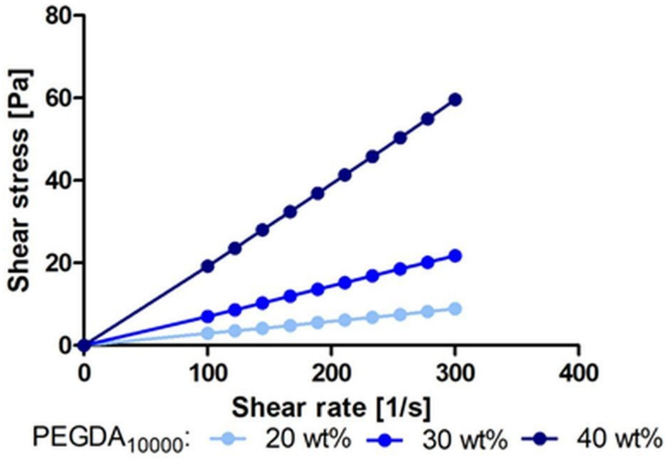

Figure S1. Exemplary shear stress/shear rate representation of $PEGDA_{10000}$ solutions in H_2O/MeOH (1:2) with different concentrations of the polymer at 15 °C (n = 3).

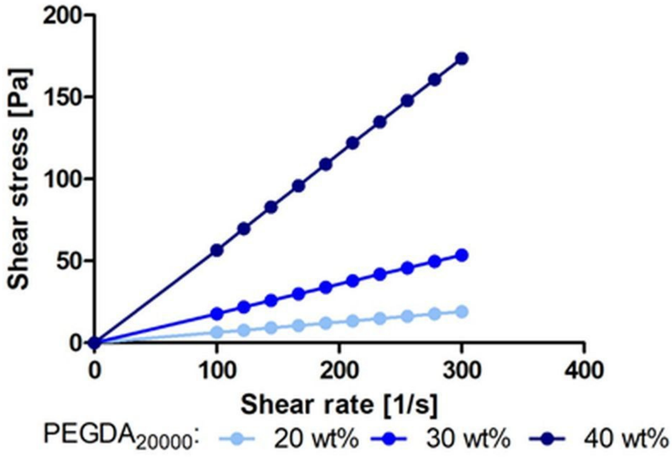

Figure S2. Exemplary shear stress/shear rate representation of $PEGDA_{20000}$ solutions in H_2O/MeOH (1:2) with different concentrations of the polymer at 15 °C (n = 3).

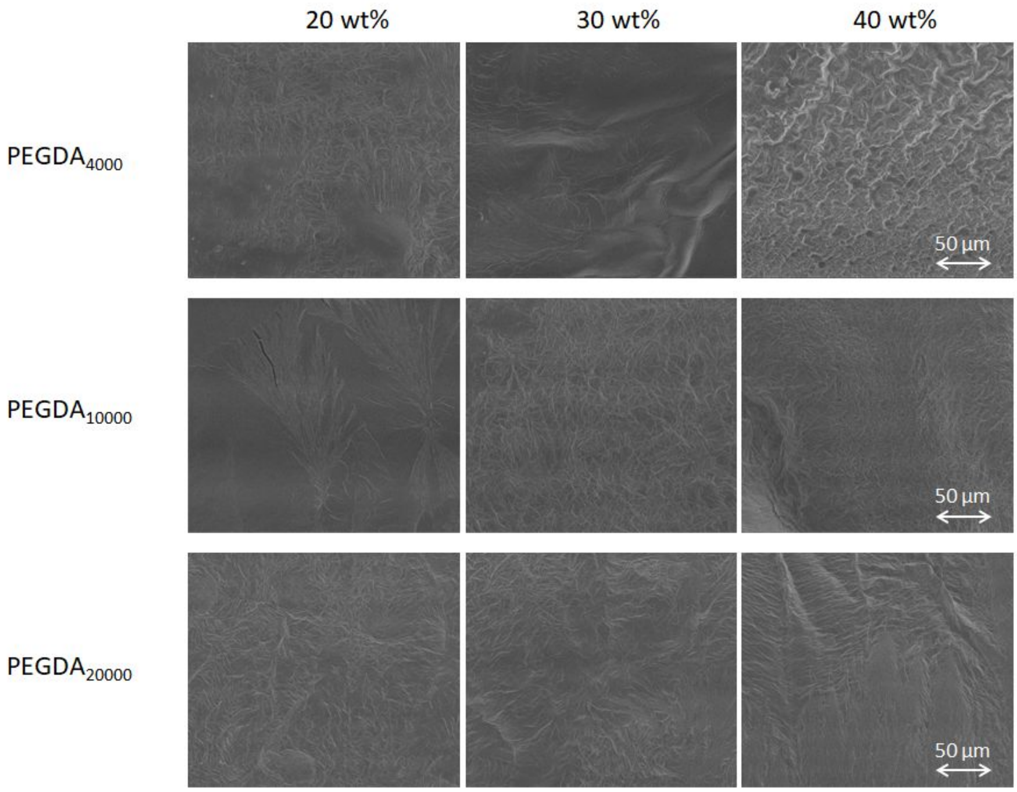

Figure S3. Comparison of SEM images of the surface of PEGDA samples with different molecular masses (4000, 10,000, and 20,000 g/mol) and their concentrations with BSA–FITC.

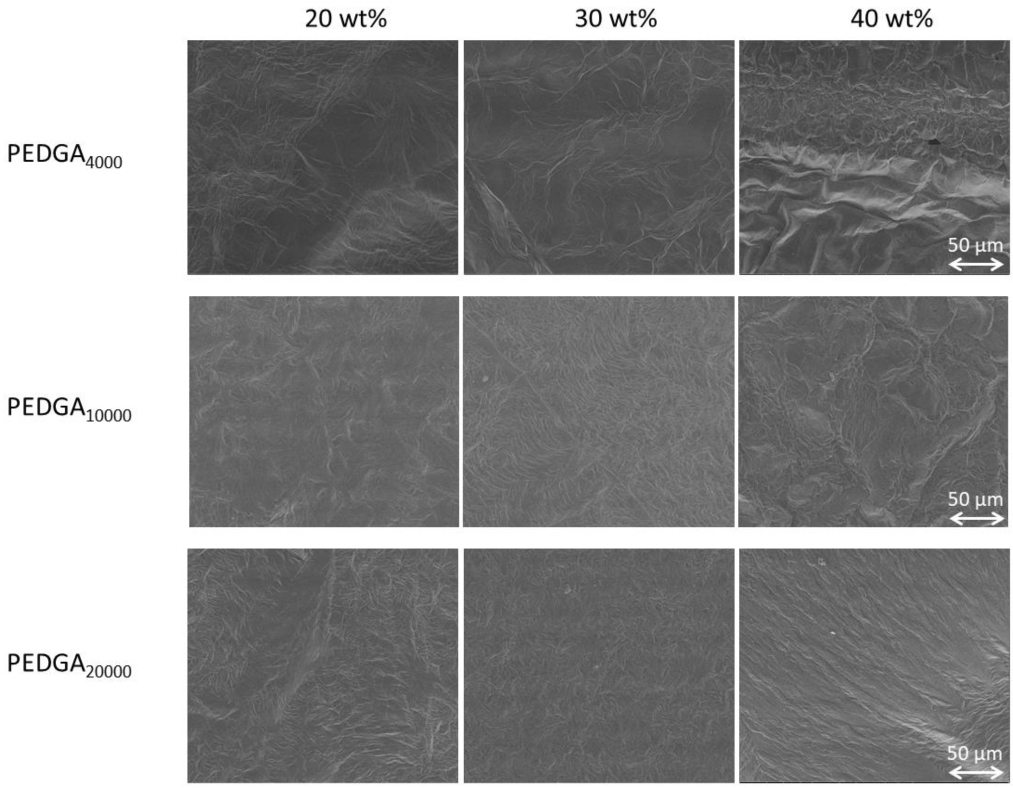

Figure S4. Comparison of SEM images of the surface of PEGDA samples with different molecular masses (4000, 10,000, and 20,000 g/mol) and their concentrations without BSA–FITC.

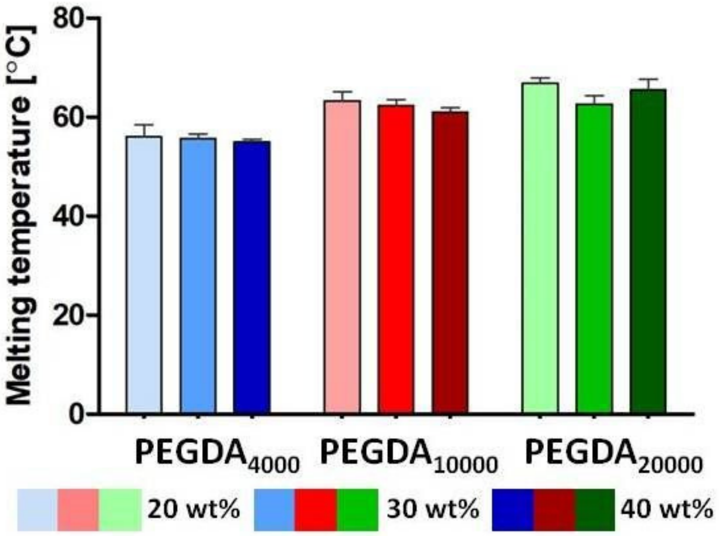

Figure S5. Mean Melting temperature[°C] with SD for $PEGDA_{4000}$, $PEGDA_{10000}$ and $PEGDA_{20000}$ samples containing 20, 30, or 40 wt % of the polymer without BSA-FITC (n = 3).

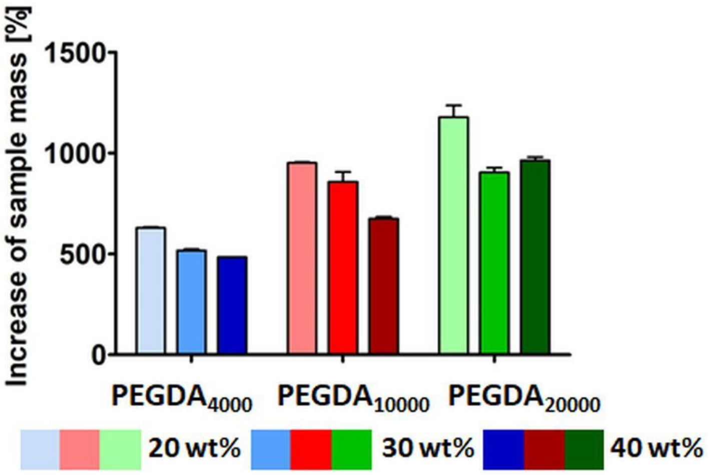

Figure S6. Increase in sample (Ø = 6 mm, thickness = 1 mm) weight (pure PEGDA without BSA-FITC) in percentage with SD after 24 h swelling in a TES buffer at 37 °C (n = 3).

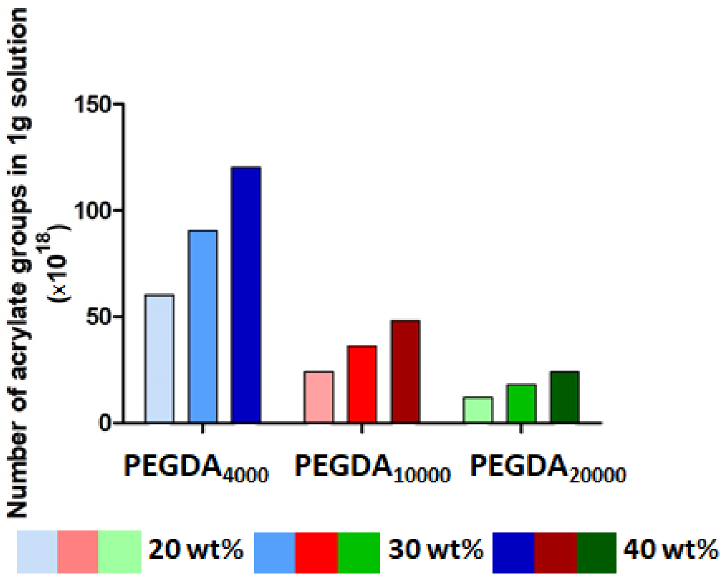

Figure S7. Theoretical number of free acrylate groups in 1 mL of unpolymerized of different PEGDA solutions.

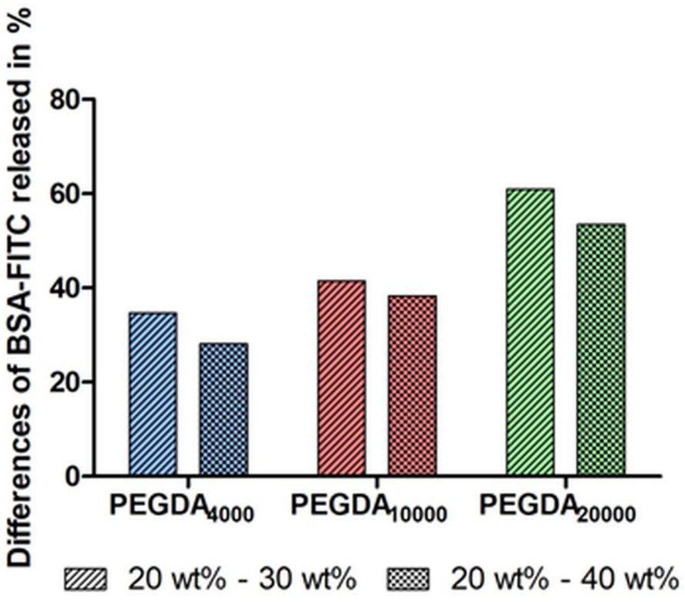

Figure S8. Differences between the 20–30 and 20–40 wt % PEGDA samples in the released amount of BSA–FITC.

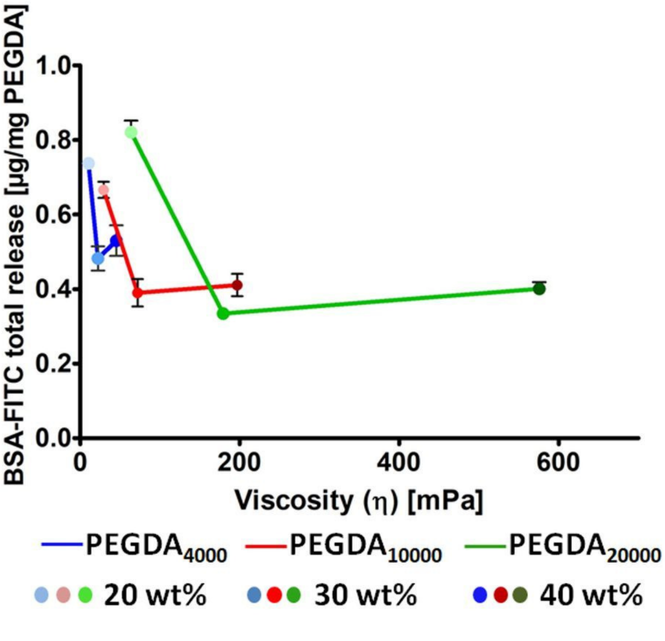

Figure S9. Relationship between total BSA–FITC release and PEGDA viscosity (4000, 10,000, and 20,000 g/mol) solutions (20, 30, and 40 wt %).
